# Cardiac auscultation training of medical students: a comparison of electronic sensor-based and acoustic stethoscopes

**DOI:** 10.1186/1472-6920-5-14

**Published:** 2005-05-09

**Authors:** Henning Høyte, Torstein Jensen, Knut Gjesdal

**Affiliations:** 1 University of Oslo, Faculty of Medicine, Oslo, Norway; 2 Department of Cardiology, Ullevål University Hospital, Oslo, Norway

## Abstract

**Background:**

To determine whether the use of an electronic, sensor based stethoscope affects the cardiac auscultation skills of undergraduate medical students.

**Methods:**

Forty eight third year medical students were randomized to use either an electronic stethoscope, or a conventional acoustic stethoscope during clinical auscultation training. After a training period of four months, cardiac auscultation skills were evaluated using four patients with different cardiac murmurs. Two experienced cardiologists determined correct answers. The students completed a questionnaire for each patient. The thirteen questions were weighted according to their relative importance, and a correct answer was credited from one to six points.

**Results:**

No difference in mean score was found between the two groups (p = 0.65). Grading and characterisation of murmurs and, if present, report of non existing murmurs were also rated. None of these yielded any significant differences between the groups.

**Conclusion:**

Whether an electronic or a conventional stethoscope was used during training and testing did not affect the students' performance on a cardiac auscultation test.

## Background

The French physician Rene Laennec invented the first stethoscope in 1816[1]. The use of a modified version was widespread among physicians in the 1830's[2], and the currently used binaural models were designed in the 1870's. Since then a number of attempts to improve the stethoscope have been made, the most recent being the advent of electronic sound transmission. "TheStethoscope^®^" is a sensor based electronic stethoscope introduced in 1999 by Meditron (Asker, Norway) in cooperation with Welch Allyn (Skaneateles Falls, USA). The stethoscope is equipped with a pressure sensitive sensor, and the signals are converted into sound waves. It is also equipped with a volume regulator and a possibility to apply frequency filtering. The filter has three modes, enhancing low (20–800 Hz), high (200–20000 Hz) or all frequencies. It can be connected to external devices (PC/co-listening unit) allowing recording or sharing of auscultatory findings.

Electronic stethoscopes offer potential advantages compared to conventional pneumatic stethoscopes[3], and several of the features unique to electronic stethoscopes could influence the performance in cardiac auscultation. The high sound quality, the possibility of applying personal adjustments to frequency[4] and volume, and education by simultaneous auscultation could improve the performance on a cardiac auscultation test. The volume regulator could also prove beneficial to students and doctors with organic hearing problems. Electronic stethoscopes are, however, sensitive to manipulation artefacts as well as electronic and ambient noise. The sound picture from an electronic stethoscope is also quite different from a conventional stethoscope, requiring training. Thus, some of the features could possibly influence the performance negatively. The volume adjuster is step-less, which could give rise to problems when grading the intensity of murmurs. The increased sensitivity to ambient noise and noise from handling of the stethoscope could increase the report of false murmurs, and lead to inaccurate characterisation of murmurs.

The aim of this study was to compare the auscultation skills of medical students using an electronic, sensor based stethoscope with a similar group using conventional stethoscopes.

## Methods

### Study population

The trial was conducted at Ullevål University Hospital (UUH) during the autumn 2001 and spring 2002, using third year medical students at the University of Oslo. Teaching groups, each comprising 6–8 students, were randomised to use either the electronic stethoscope (intervention group) or their own acoustic stethoscopes (control group) during a four month training period. A total of 48 students were enrolled, 24 in each group.

### The teaching of cardiac auscultation

The students at the University of Oslo are introduced to cardiac auscultation during propedeutic clinical courses in the second year, and more extensively during rotations in cardiology in the third year. In addition to the regular course program the students in our trial received a two hour lecture and four hours of clinical bedside teaching. The intervention group using used simultaneous auscultation during bedside teaching.

### The auscultation test

The students' auscultation skills were tested on patients at the university hospital. Each student completed a questionnaire (mainly multiple choice questions) on auscultation findings for each patient (table 1). Next to each patient was a brief survey of the patient's presenting complaints, and the patients were instructed not to reveal their diagnoses. The students were allotted ten minutes to examine each patient. They were alone with the patients during examination, and were instructed not to discuss their findings with any other student. A total of ten patients participated, one of them twice. They were recruited either from the ward or from the clinic's outpatient population. (Table 2)

**Table 1:** The questionnaire. shows the questionnaire the students had to complete.

**Question No:**		**Alternatives**	**Points**
1	Do you hear any murmur?	Yes/no	6
2	If so, is the murmur:	Systolic/ Diastolic /both	5
3	If you have heard a systolic murmur, how would you characterise it?	Holosystolic/Crescendo-decrescendo	2
4	Describe the quality of the systolic murmur:		3
5	Where is the murmur loudest?	Anatomical alternatives	3
6	Grade	1 – 6	4
7	Radiation?	Anatomical alternatives	4
8	If you have heard a diastolic murmur, how would you characterise it?	Rumbling, whistling etc.	3
9	Is the 2^nd ^heart tone preserved?	preserved/ diminished/ not audible	4
10	Is the 2^nd ^heart tone constantly split?	Yes/ no	1
11	Is a third heart tone present?	Yes/ no	1
12	What is the most likely cause of the murmur?	Options	2
13	Any comments?		Max 2

**Table 2:** The patients' diagnoses and findings on auscultation. shows the patients relevant cardiac diagnoses, and the findings on auscultation as reported by two cardiologists.

**Patient nr 1**	**Diagnoses**	Findings*
***Day 1: ***Patient no 1 (1)	Mitral valve insufficiency, possible low grade aortic sclerosis	Holosystolic, rough high frequency, grade 3/6, max intensity in the fourth left intercostal space in the medioclavicular line with radiation to the left axilla. 2^nd ^heart sound (S2) preserved, no constant splitting of S2. S3 not present.
Patient no 2 (2)	Aortic stenosis	Holosystolic rough murmur grade 4/6, loudest at the apex. Radiation to the left axilla and the carotid arteries. S2 weakened.
Patient no 3 (3)	Control patient without cardiac murmurs	-
Patient no 4 (4)	Aortic insufficiency	High frequency diastolic murmur distinct at the apex.
***Day 2: ***Patient no 1 (1)	Same as day 1	Same as day 1.
Patient no 2 (5)	Control without murmur	-
Patient no 3 (6)	VSD	Holosystolic ejection sound grade 3/6, loudest at the apex. No radiation. S3 present.
Patient no 4 (7)	Mitral valve insufficiency and aortic sclerosis	Holosystolic ejection sound grade 4/6. loudest at the apex, radiation to the left axilla and the carotid artery. S3 absent.
***Day 3: ***Patient nr 1 (8)	St. Jude prosthetic valve in mitral position, paravalvular leakage	Holosystolic rough murmur grade 2, loudest in the left axilla.
Patient nr 2 (9)	Control without murmurs	-
Patient no 3 (10)	Aortic stenosis	Crescendo/decrescendo quincking systolic murmur grade 3/6, loudest parasternally at the right second intercostal space. Radiation to the left axilla and to the carotid arteries.

*The findings on auscultation as reported by the two cardiologists.

### Scoring

The correct answers on the questionnaire were defined by consensus of two cardiology consultants who examined the patients with acoustic stethoscopes on the same day as the students were tested. Each questionnaire was interpreted and scored blindly by one person. When there was doubt about scoring, the questionnaire was in addition evaluated by a second person, and consensus was reached. A correct response to each of the questions was rewarded by a predefined number of points, ranging from one to six (table 1). The points obtained on each question were added, a total score for the questionnaire calculated, and total and average scores were obtained for each group.

Based on our own experiences with this electronic stethoscope, we also wanted to test whether there were differences between the two groups' regarding grading and characterising murmurs, and report of non-existing murmurs.

Different scores were allotted for each patient and the mean max score that could be obtained differed. For all days combined the mean maximum score was 25.3 points.

### Statistics

Data are reported as means with confidential intervals (CI) or range. Differences between the study groups were evaluated using Student's t-test. When comparing categorical data Chi-square tests were used. Calculations regarding group size and statistical power were done in retrospect. The reason for this was the difficulty of estimating the standard deviation (SD) prior to the trial. Each group comprised 24 students, providing 80% power of detecting a difference of seven points between the study groups (SD = 8.6). P-values are two-sided, and values <0.05 are regarded significant.

## Results

Each student contributed three or four questionnaires (depending on the day of participation). Forty-one of the students (85%) completed the trial. (Two had exrolled from the faculty, one was abroad, and four did not meet for other reasons. Four of these students were from the group using the electronic stethoscope.) Three questionnaires were incomplete and excluded from final analysis.

**Figure 1:**
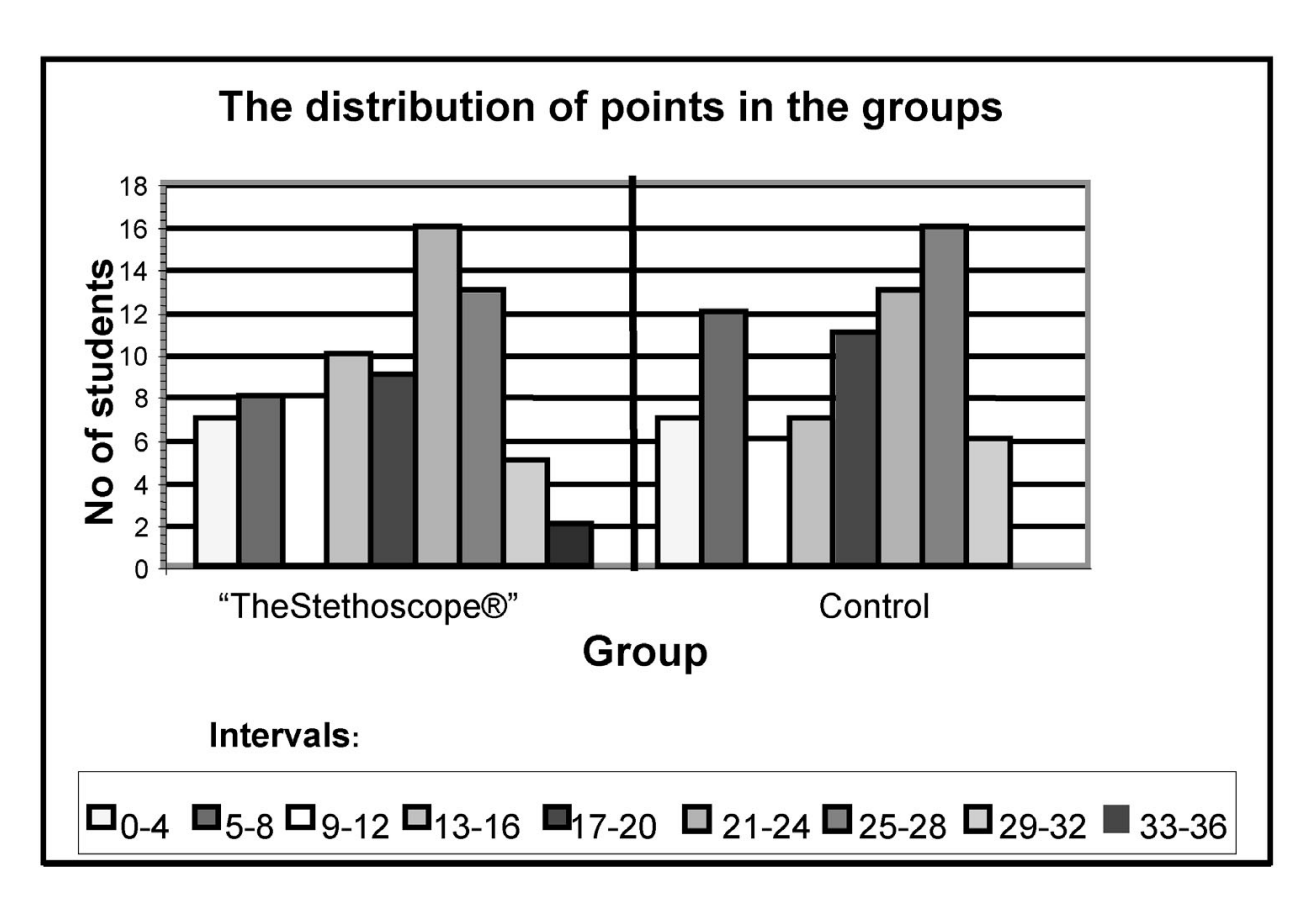
The number of students distributed on intervals of points.

The number of questionnaires scored was 78 and equal in the two groups. The total score in the control group was 1341.5 points versus 1388.5 in the intervention group. Mean scores in the control group and intervention group were 17.2 (SD = 8.7, range 1–30.5) and 17.8 (SD = 8.8, range 0–35) points respectively. The difference is 0.6 points with a 95% CI of (-0.33 – 1.53) points (p = 0.65). (Figure 1)

When grading the murmurs, the students using conventional stethoscopes had 27 correct and 27 incorrect responses, whereas the students using the electronic stethoscope had 29 correct and 22 incorrect responses (p = 0.47). On characterising murmurs the group using conventional stethoscopes had 45 correct and 66 incorrect responses, while the students using the electronic stethoscope had 42 correct and 66 incorrect responses (p = 0.76). When tested for report of false murmurs, the group using conventional stethoscopes had 12 correct and 8 incorrect responses. The group using the electronic stethoscope had 11 correct and 9 incorrect responses (p = 0.75). (Table 3)

**Table 3:** Overview of the results in the two groups. Per cent correct answers. The results in the intervention and control group reported as % correct answers.

	Electronic stethoscope	Control
Total score (mean ± SD)	17.8 ± 8.8	17.2 ± 8.7
Grading of murmurs, % correct	29 correct of 51 answers; 43%	27 correct of 54 answers; 50%
Characterization of murmurs, % correct.	42 correct of 109 answers; 39%	45 correct of 111 answers; 41%
Report of false murmurs, % correct.	11 correct of 20 answers; 55%	12 correct of 20 answers; 60%

## Discussion

The aim of our study was to determine if the use of an electronic stethoscope would influence cardiac auscultation skills of undergraduate medical students. To investigate this we compared the performance on a cardiac auscultation test of a group of medical students using conventional stethoscopes to a group using electronic stethoscopes. No differences between the study groups were found, neither for general nor for more specific skills in cardiac auscultation, such as grading and characterising murmurs, and report of non existing murmurs. We are not aware of any similar studies comparing electronic and acoustic stethoscopes.

Several factors strengthen our results. We used a prospective, randomized study design with test conditions nearly similar to a regular clinical setting. The opportunity to gather anamnestic information was limited, as we did not want any of the patients to reveal their diagnoses. A note, however, next to each patient summarised the relevant clinical data. Thus the students received exactly the same information. 85% of the included students completed the test, which is a high percentage considering the logistic difficulties inherent in use of patient and student volunteers.

The results in the two groups are strikingly similar, both for the main (figure 1) and for the additional hypotheses tested (table 3). It is therefore unlikely that the limited size of the groups has introduced a type II error. The size of the groups gives an 80% chance of discovering a difference between the groups of seven points. A difference of seven points is quite much, 40% of the average total score. The question can be raised if detection of a smaller difference between the groups would be clinically relevant.

It can be objected that our diagnoses were based on auscultation and not verified by echocardiography. However, we were primarily testing auscultatory findings and not diagnostic interpretation. We justify the use of the cardiologists' auscultatory findings as a gold standard for the students since one should not expect that the students would have greater auscultatory proficiency than the cardiologists [5-7]. Some of the patients used in the auscultation test were, however, known to the cardiologists, and there is a possibility that their findings on auscultation could be biased by background information about these patients.

Simultaneous auscultation was used as an additional teaching tool in our study. The instructors did not, however, receive any special training in the use of this new technique. This might have limited the opportunity to get full effect of this method. It is also possible that the intensity of the teaching intervention was insufficient, and that additional hours of teaching using simultaneous auscultation would have improved the cardiac auscultation skills selectively.

We chose to test our main hypothesis using a system of points for each question. Wrong answers on questions testing the most important auscultatory skills lead to a loss of most points, whereas incorrect answers on questions testing more advanced skills were punished less[8]. As an example a student who was unable to separate a systolic from a diastolic murmur lost five points, whereas a student missing a third heart tone lost only one point[9]. A correct diagnosis gave only two points, as this also tests other skills not related to the stethoscope used. The weighting of the questions was done prior to the test. We attempted to accommodate what we could expect from students at this level and that we tested the students' ability to report auscultatory findings, not their skills in general clinical examination.

When using points to grade the question it is of importance that the groups are evenly distributed on the patients. Not all the questions are applicable to all the patients, and the maximum number of points achievable varied between the patients (table 4). As seen in table 5, the two groups are evenly distributed on the test days and thus on our test patients.

**Table 4:** The mean scores in the groups for the different patients.

Patient no.	1	2	3	4	5	6	7	8	9	10
Mean scores:										
"The Stethoscope^®^":	20	26	7.6	18.9	5.8	19	27	14.5	7	21
Control group:	21.1	26	4.8	18.4	6.8	17	24	10.7	7.3	27

**Table 5:** The distribution of the groups on the test days. Shows the number of students present from each group the different test days.

**Group**	**Day 1**	**Day 2**	**Day 3**
"The stethoscope^®^"	n = 7	n = 12	n = 1
Control	n = 5	n = 12	n = 4

Each student was represented by three or four questionnaires (depending on the day of participation), and each questionnaire was treated as an independent variable in the statistical analysis. This is likely to underestimate the spread in the groups, but the averages, and thus the comparison of the two groups, are not affected.

The students received the electronic stethoscopes four months prior to the auscultation test. This should be sufficient time to get accustomed to the electronic stethoscope, although it is possible that a longer period is needed to take full advantage of the additional features. It is also possible that the students' skills in cardiac auscultation are insufficient to reveal an existing significant difference between the stethoscopes. There is, however, no available documentation that cardiologists perform better with electronic compared to conventional stethoscopes, but it could be of interest to investigate if this could be the case.

## Conclusion

The cardiac auscultation skills of undergraduate medical students were not influenced by the use of an electronic sensor-based stethoscope.

## Competing interests

The author(s) declare that they have no competing interests.

## Authors' contributions

HH participated in the design of the study, the acquisition of data, performed the statistical analysis, drafted the manuscript. KG and TJ concieved the study, contributed to the design, acquisition of data and the preparation of the manuscript.

All authors read and approved the final manuscript.

## Pre-publication history

The pre-publication history for this paper can be accessed here:


